# Enhanced sensing and conversion of ultrasonic Rayleigh waves by elastic metasurfaces

**DOI:** 10.1038/s41598-017-07151-6

**Published:** 2017-07-28

**Authors:** Andrea Colombi, Victoria Ageeva, Richard J. Smith, Adam Clare, Rikesh Patel, Matt Clark, Daniel Colquitt, Philippe Roux, Sebastien Guenneau, Richard V. Craster

**Affiliations:** 1 Department of Mathematics, Imperial College London, South Kensington Campus, London, UK; 2 Optics and Photonics, University of Nottingham, Nottingham, UK; 3 Department of Mechanical, Materials and Manufacturing Engineering, University of Nottingham, Nottingham, UK; 4 Department of Mathematical Sciences, University of Liverpool, Liverpool, UK; 5 ISTerre, CNRS, Univ. Grenoble Alpes, Grenoble, France; 6 Aix Marseille Univ, CNRS, Centrale Marseille, Institut Fresnel, Marseille, France

## Abstract

Recent years have heralded the introduction of metasurfaces that advantageously combine the vision of sub-wavelength wave manipulation, with the design, fabrication and size advantages associated with surface excitation. An important topic within metasurfaces is the tailored rainbow trapping and selective spatial frequency separation of electromagnetic and acoustic waves using graded metasurfaces. This frequency dependent trapping and spatial frequency segregation has implications for energy concentrators and associated energy harvesting, sensing and wave filtering techniques. Different demonstrations of acoustic and electromagnetic rainbow devices have been performed, however not for deep elastic substrates that support both shear and compressional waves, together with surface Rayleigh waves; these allow not only for Rayleigh wave rainbow effects to exist but also for mode conversion from surface into shear waves. Here we demonstrate experimentally not only elastic Rayleigh wave rainbow trapping, by taking advantage of a stop-band for surface waves, but also selective mode conversion of surface Rayleigh waves to shear waves. These experiments performed at ultrasonic frequencies, in the range of 400–600 kHz, are complemented by time domain numerical simulations. The metasurfaces we design are not limited to guided ultrasonic waves and are a general phenomenon in elastic waves that can be translated across scales.

## Introduction

Elastic rainbow phenomena, capable of spatially selecting surface wave packets, based upon frequency, have recently been demonstrated numerically in connection to seismic metamaterials^1^. In contrast to acoustic^2–4^ and electromagnetic^5^ situations, where the main advantage of the rainbow effect is the spatial segregation of waves accompanied by the local enhancement of their amplitude, the elastic rainbow for elastic surface waves has an additional remarkable property which is that of broadband mode conversion from Rayleigh surface waves into bulk shear waves.

Rainbow devices combine the remarkable properties and capabilities of locally resonant metamaterials, such as subwavelength bandgaps and trapping^6–9^, with convenient graded or chirped structures. Graded designs take advantage of slow sound (or slow light), generated by spatial graduation, progressively decreasing the group velocity of waves they support. This creates media with spatially varying refraction index that find wide application in phononics (or photonics) because of their excellent abilities to manipulate and filter waves in compact devices^10–16^. Wave control with a graded design does not always rely upon resonant phenomena; spatially varying index material for, say, gradient index (GRIN) lenses^17^ can alternatively, and more easily, be obtained through graded composite media^18–21^. However the use of local resonators, as we propose here, gives access to the very subwavelength scale and the power to have precise control over long waves.

For instance, in rainbow trapping, the combined graded and resonant structure allows waves to be slowed down selectively upon their frequency, and eventually to be trapped in a subwavelength area, producing a strong local enhancement of their amplitude^2, 3, 5, 22^. This enhancement is attracting most of the attention as it can positively impact technologies for energy harvesting, sensing and absorption of waves in electromagnetism^23, 24^, acoustics^25, 26^, and, as discussed here, elastodynamics^1^.

Despite the success of these ideas, there have been surprisingly few applications of chirped and graded resonant structures to mechanical metamaterial devices. However, dramatic improvements in fabrication techniques (additive manufacturing, self-assembly and 3D printing), mean that the complexity of smoothly varying designs can now be easily realised. For instance, only recently this has been considered for GRIN lenses^17^ for flexural waves using resonators on an elastic plate^15^ and, in the context of rainbow devices, by using chirped graded array on a plate^22^ and thin beams with tunable piezoelectric patches^27^. A key simplification of thin elastic plates (or thin beams) is that the aspect ratio of plate thickness *b* to wavelength *λ* is limited to $b\ll \lambda$ and low frequency flexural (*A*_0_) waves are dominant. This is not the case for deep elastic substrates that support both bulk compressional and shear waves^28^, with different wavespeeds, moreover both wavetypes couple and mode convert at interfaces with the Rayleigh surface wave^29^. Because of the lack of this wave polarization in acoustics or flexural thin plates, recent experiments with acoustic waves^2, 26^ in graded grooved waveguides and graded elastic plates^22, 27^ do not support mode conversion and can only demonstrate trapped rainbow phenomena. On the other hand full elastic models taking account of graded subwavelength resonator arrays have only just appeared and were recently envisaged at the low frequencies (10~50 Hz) and large-scale (tens or hundreds of metres) associated with geophysical applications^1^. A key claim of this theoretical work is that one not only has elastic wave trapping and Rayleigh wave rainbow phenomena due to mechanisms described earlier, but one can in addition engineer mode conversion from surface to bulk modes. Furthermore, it is claimed that this mode conversion can create a surface array that is simultaneously reflectionless (as opposed to the rainbow trapping), yet has zero transmission into surface waves, and moreover that this occurs over an ultra-broadband range of frequencies.

In the following, we move the entire theory from seismic to ultrasonic frequencies, and scales from decametres to millimetres, to design and build experiments that conclusively demonstrate both elastic Rayleigh wave rainbow trapping and mode conversion phenomena. We develop the physical interpretation of these observed results through fully 3D time dependent elastic numerical simulations and Bloch-Floquet theory for resonant elastic halfspaces^30^. Finally we discuss some potential applications of Rayleigh trapping and conversion in the context of vibration absorption, signal filtering, energy harvesting and enhanced sensing.

## Designing the graded subwavelength array

**Figure 1:**
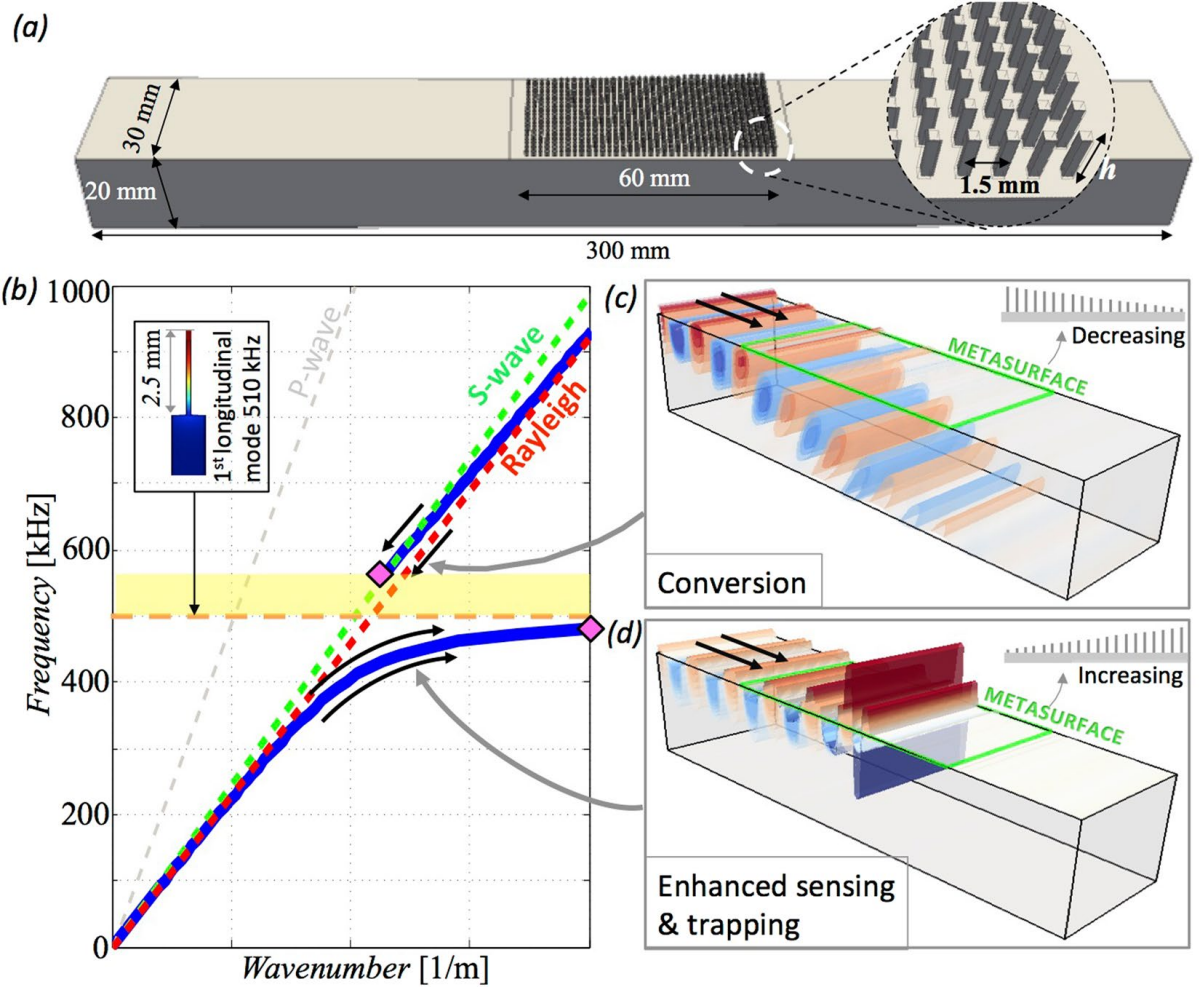
(**a**) Design and dimensions of the metasurface discussed in this study. (**b**) The dispersion curves of an elastic halfspace for ultrasonic frequencies measured at the top surface featuring surface Rayleigh (red) and shear (green) *S*-waves. The *P*-wave dispersion is marked with a dashed grey line. The introduction on the surface of local vertical resonators, (shown in the inset with the corresponding modal deformation), modifies the dispersion properties introducing a hybrid curve (blue) characterised by a bandgap (yellow area) bounded from below by an ultra slow mode and from above by a fast wave (pink diamonds). The *S*-wave serves as the light line, limiting the maximum speed of this system. (**c**) When Rayleigh waves approach an array of resonators of decreasing height (increasing resonance frequency), they are smoothly converted into *S*-waves. (**d**) Conversely, when the resonators are of increasing height, waves are spatially segregated, amplified and reflected.

Figure 1a sketches the metasurface configurations: an aluminium block of rectangular cross-section (20-mm-thick, 40-mm-wide and 300-mm-long) with an array of decreasing or increasing resonators centred on the top surface; as discussed in the methods section, the array of vertical rods is fabricated on top of the block using precision milling from a single metal block. The orientation of the graded array with the shortest, or longest, resonators facing the incident field determines whether Rayleigh wave trapping (Fig. 1c) or mode conversion occurs (Fig. 1d); the mechanical properties of aluminium are summarised in Table 1.

**Table 1:** Resonator parameters for Fig. 1b (constant height, cross-sectional area and spacing) and the mechanical properties of aluminium.

Height *h* [mm]	Section *A* [mm^2^]	Spacing *s* [mm]	Young modulus*E* [GPa]	Density *ρ* [kg/m^3^]
2.5	0.025	1.5	69.0	2710.0

To clearly interpret the physics, the characteristics of the metasurface are initially explored considering an infinite periodic array of resonators, of identical height (see inset in Fig. 1b and rod’s properties Table 1). Periodicity enables us to utilize Bloch theory to consider a single resonator in a cell with Floquet-Bloch condition upon the vertical edges of the cell. The resulting dispersion curve (blue line) relating phase shift across the cell to frequency is calculated using a 2D analytical approach that couples the longitudinal motion of the microrods with the full Navier elasticity equations in the halfspace^30^. The resulting shape of the dispersion curve is characterised by “hybridization” between the out-of-plane component (*u*_*z*_) of the Rayleigh wave and the fundamental longitudinal mode in the rods^7, 31, 32^. Notably, in this framework, flexural modes within the rods have negligible effect and can be ignored.

The dispersion properties of the metasurface strongly differ from that of the bare aluminium block for which we show the dispersionless sound-lines associated with compressional *P*-waves (grey dashed), shear *S*-waves (dashed green) and surface Rayleigh waves (dashed red line). The resonance frequency (orange dashed) line punches through the sound-lines and, as is well-known, this perturbation leads to dispersion curve repulsion related to eigenvalue avoided crossing^33, 34^, and the creation of a band-gap (yellow shaded zone). It is clear that the array of constant height resonators is extremely powerful in terms of stopping (bandgaps, yellow shaded zone) and modifying the Rayleigh wave group velocity (flat branches for high wavenumbers). However the broadband performance is poor as control is achieved only around resonance that, in this case given the height of the aluminium microrod (*h* = 2.5-mm), is located at ~510 kHz (dashed horizontal line). Graded metamaterial designs (e.g. Fig. 1c and d) overcome this limitation in bandgap size to create ultra-broadband devices by introducing more degrees of freedom to engineering dispersion curves; since the resonators underpin the dispersion properties, their dynamic response can be tuned to build metamaterials with spatially graded properties^15^. For the microresonator considered here, the fundamental longitudinal mode frequency *f* is simply defined as:

1 $$f=\frac{1}{4h}\sqrt{\frac{E}{\rho }},$$

where *h* is the resonator height, *E* its Young’s modulus and *ρ* its density. In this formula longitudinal resonance is inversely proportional to the resonator height, *h*, that is then chosen as the tuning parameter for the metasurface in Fig. 1c and d. Despite the complexity added by the graded design, Eq. (1), complemented with the dispersion diagram in Fig. 1b, is sufficient to fully describe the rainbow trapping and to reveal a unique feature associated with elastic waves: the conversion of surface Rayleigh into bulk shear *S*-waves. Rainbow trapping is generated by the nearly flat branch approaching zero group velocity at the edge of the Brillouin zone, while the conversion is due to a hybrid mode, typical of this metasurface, connecting the Rayleigh with the *S*-wave line. Both phenomena are functions of the resonance frequency of the microrods and for the proposed designs in Fig. 1c and d, the trapping and turning points are precisely predicted using Eq. (1) (see later discussions). When considering the propagation of Rayleigh waves, the bandgap (yellow region Fig. 1b) yields a discontinuity on the hybrid curve (pink diamonds in Fig. 1b). Thus, the lack of a propagative solution for the surface wave in this region means that waves, depending on which branch of the blue line they approach, can either be stopped and reflected back or converted, away from the surface, as bulk *S*-waves. The discontinuity also determines the non-reciprocal behaviour of the metawadge that acts as a barrier between two states. For the increasing height case, we only obtain trapping (Fig. 1b, arrows following the flat branch), while the decreasing case can only lead to conversion (Fig. 1b, arrows pointing towards the *S*-line)^1, 30^.

**Figure 2:**
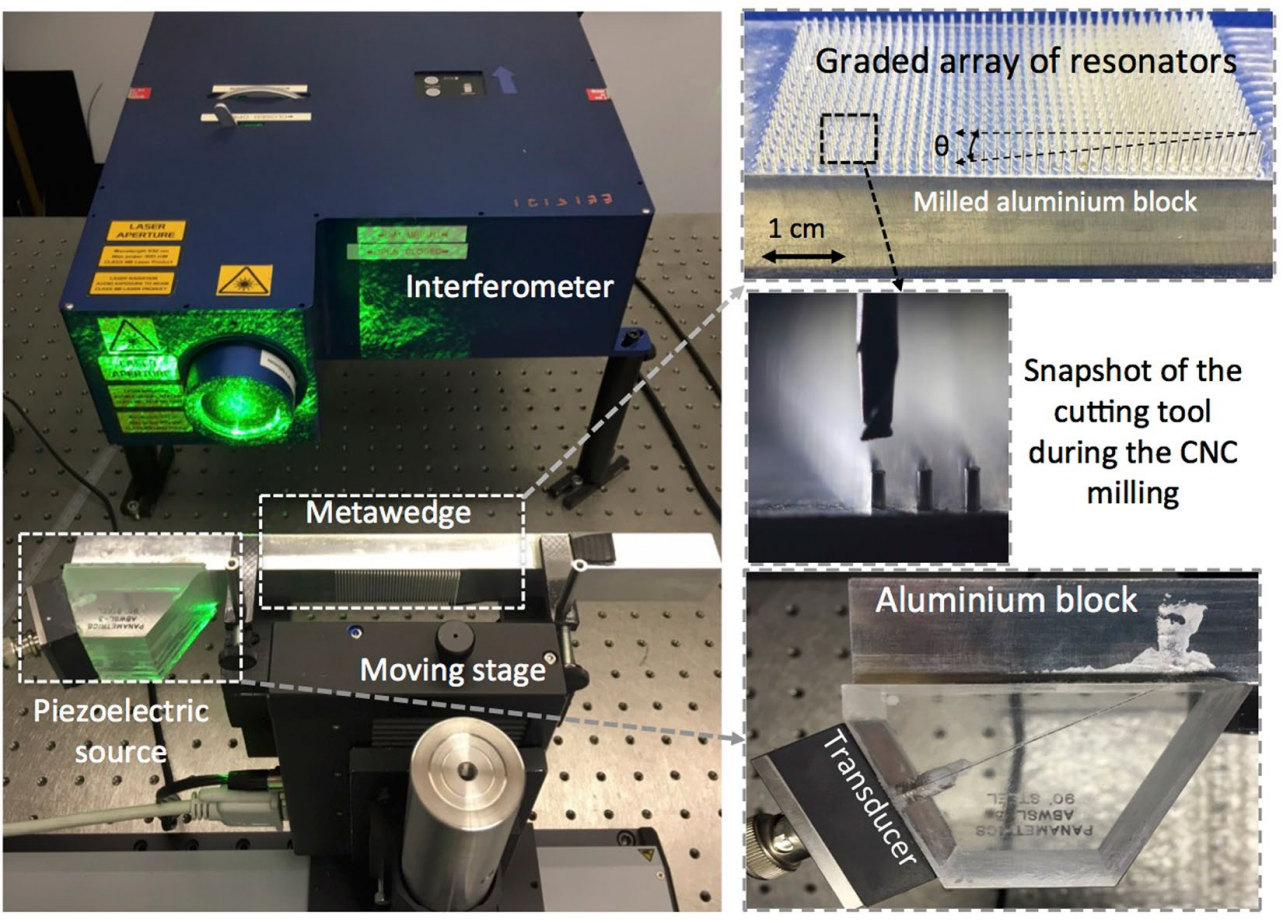
The experimental set-up consisting of a laser interferometer scanning the surface of the metawedge and the aluminium block mounted on a moving stage. The excitation is obtained with an ultrasonic transducer coupled with a 65° wedge that, by phase matching, filters out all incident waves except Rayleigh waves. The resonators have a 0.5 × 0.5-mm cross-section and an increasing (or decreasing) height from 0.5-mm to 3-mm and have been obtained by micro-milling of the surface.

Using the interpretation and modelling, the metawedge design chosen consists of a 1.5-mm spaced array of 40 rows each containing 18 microresonators with wedge height varying with an angle *θ*~2.5° (more details in Figs 1 and 2 and in the Methods section) from 3 mm the tallest to 0.5 mm the shortest. The finite size of the aluminium block, and the presence of free surface boundaries at the surface of the block, leads to small reflections whose effect is quantified by analysing 3D numerical simulations of the experimental set-up implemented using the spectral element solver SPECFEM3D^35^ (see Methods).

### Elastic rainbow and mode conversion

**Figure 3:**
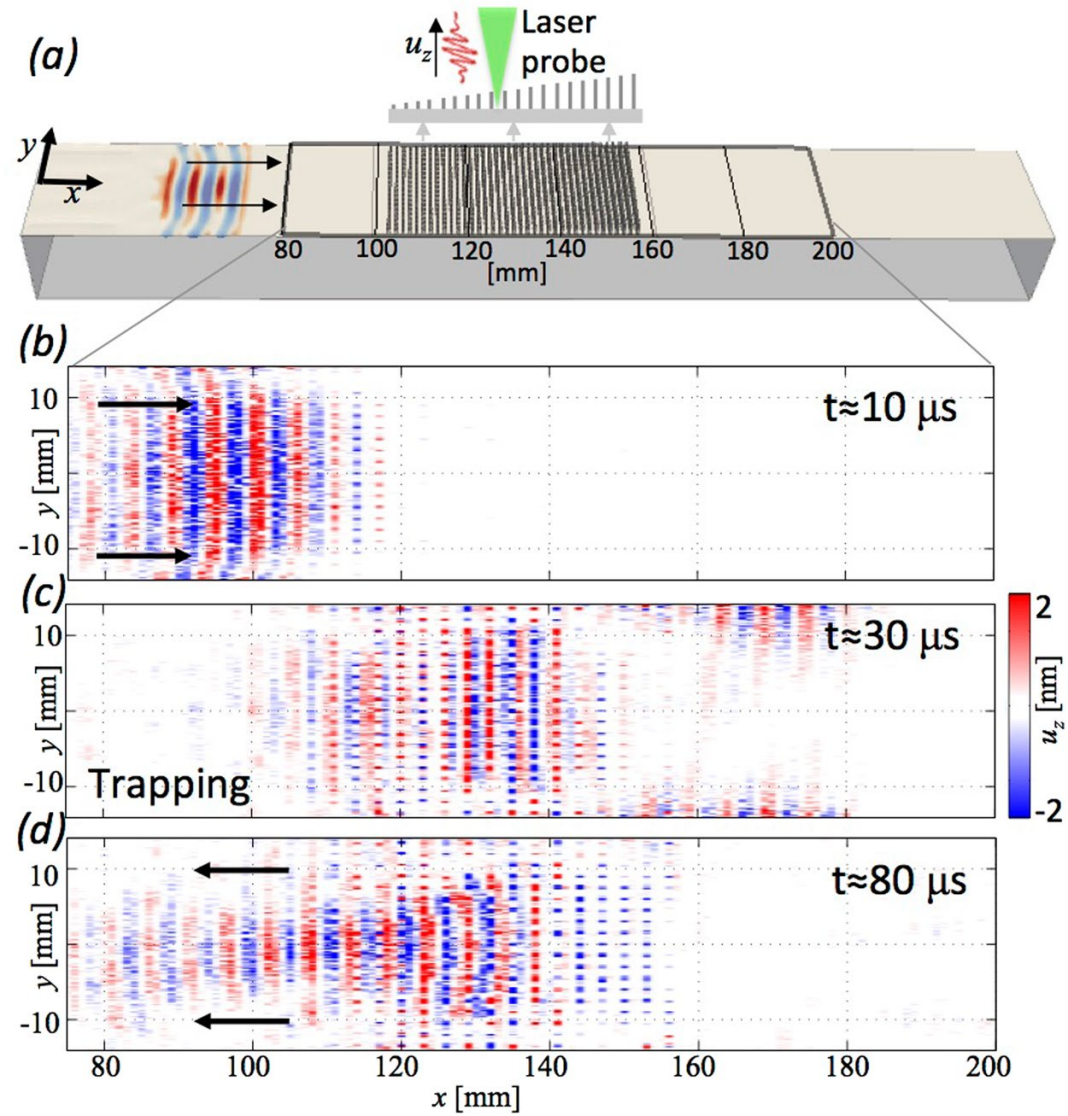
Experimental results for an increasing wedge profile. Data have been filtered between 450 and 650 kHz to improve the visualisation. (**a**) The numerical representation of the experiment. (**b**) The train of surface Rayleigh waves before entering the wedge, (**c**) trapped inside the wedge and (**d**) reflected backward. The underside of the sample was also scanned and no wave was found (see Fig. 5 and supplementary videos).

**Figure 4:**
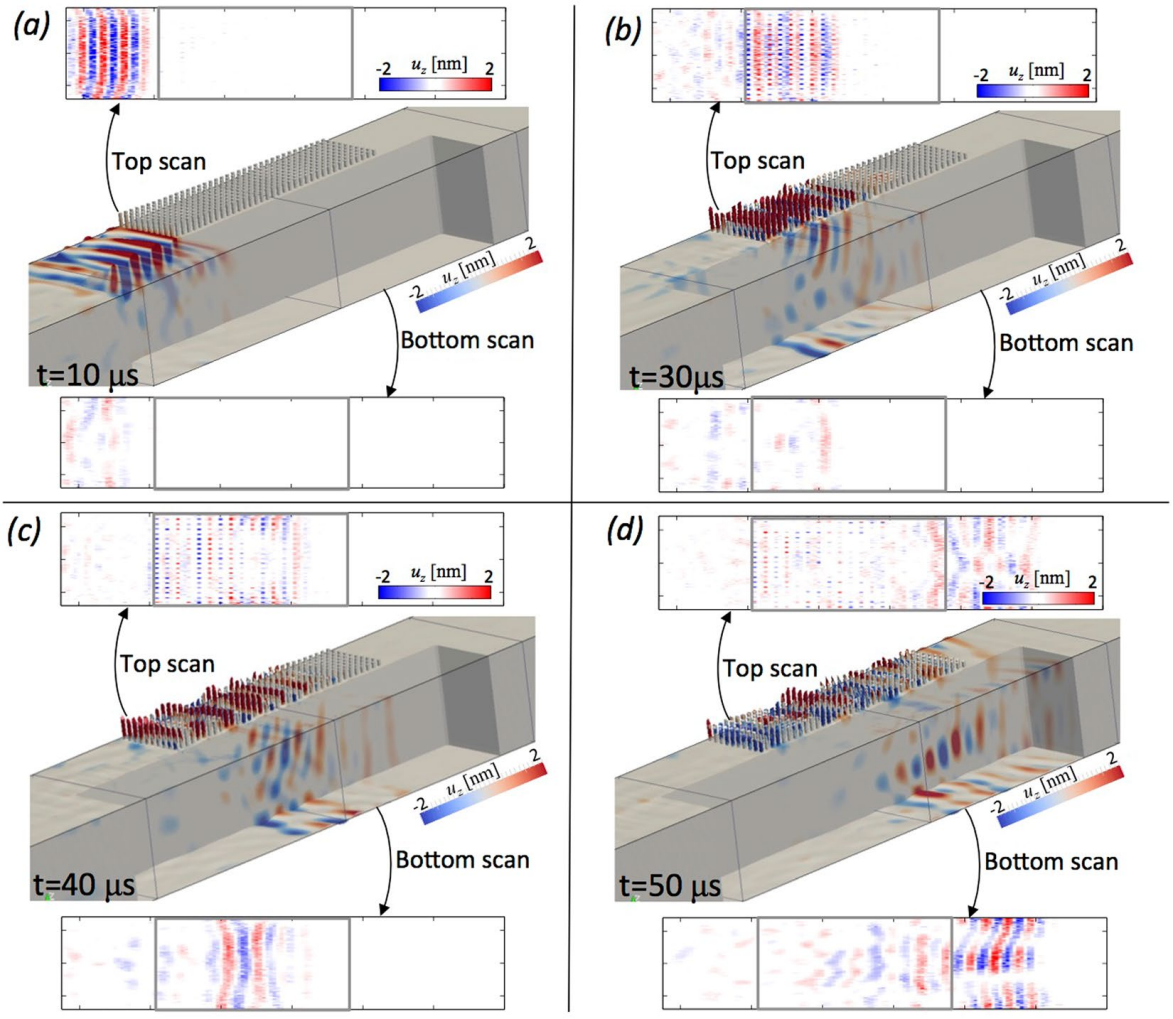
The conversion of surface Rayleigh into shear, *S*-waves seen both from the numerical and experimental perspective. (**a**) The top and bottom surface scans from the experiment are shown together with the corresponding time-frame of a 3D numerical simulation reproducing the experiment. (**b–d**) same as (**a**) but for later time-steps.

Once fabricated, the metawedge was mounted on a moving stage as shown in Fig. 2; a laser adaptive photorefractive interferometer scans the surface of the block and records the displacement field in the out of plane direction *u*_*z*_. An ultrasonic transducer generates, via phase matching with a 65° wedge, purely Rayleigh waves at the surface of the aluminium block. The input signal consists of a 3 cycle sinusoid propagating towards the metasurface (Fig. 3a). The signal excites frequencies between 400 and 600 kHz with a maximum around 500 kHz (see further details in the last section). The scanning procedure is repeated iteratively with the laser focal point moved around the block surface on a 2D grid (see Methods for details on the experimental set-up). To validate experimentally the Rayleigh to *S* wave conversion the back surface is also scanned (Fig. 4). Given the type of source and the experimental settings the surface displacement is typically of the order of a few nanometres.

We demonstrated rainbow trapping by launching a Rayleigh wave at the metawedge from the short side (see the configuration in Fig. 4a). The train of Rayleigh waves approaches the metawedge interacting with the shortest resonators first and the interferometers scans the top surface. The snapshot in Fig. 4a, extracted from a 3D numerical simulation, shows that the plane wavefront is only marginally affected by the finite nature of the aluminium block and the Rayleigh waves primarily propagate as plane waves. The surface displacement scans in Fig. 3b–d show respectively the first interaction with the metasurface, the rainbow trapping and finally the reflected wavefield moving backward (movie available as supplementary material). The underside of the sample was also scanned and no wave was found. The elastic rainbow effect spatially segregates the broadband signal based upon the frequency inside the metasurface, hence low frequency waves propagates longer in the metawedge while high frequency are trapper earlier. As a result of this frequency dependent phenomenon, the backward propagating field in Fig. 3d has lost the signal spatial coherence that characterised the input wavefield in Fig. 3b. Some energy leaks out of the metasurface because of waves guided by the lateral boundaries (Fig. 3c). Notably, during the trapping, the wave is strongly slowed down and the amplitude magnitude saturates the colorscale.

**Figure 5:**
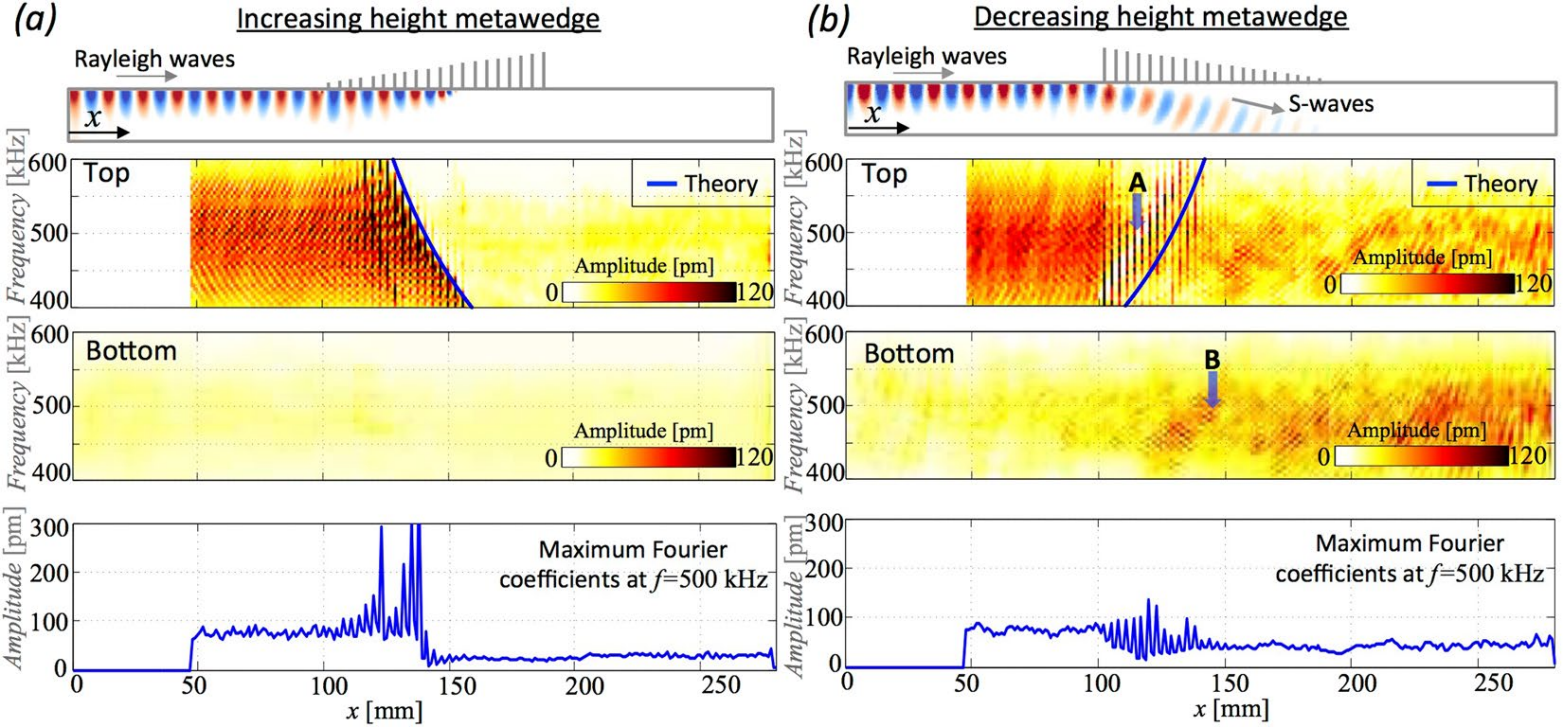
(**a**) Space-frequency analysis of the experimental data (displacement) for the increasing metawedge. From top to bottom: the geometry, and field. The next two panels show the power spectra along the *x*-direction in the aluminium block measured at the centreline for the top and bottom surfaces. The white area in the surface plot is due to the transducer. The lowest plot represents the maximum value of the Fourier coefficients at 500 kHz for the scan located approximately at the centre of the top surface (*y* = 0). (**b**) Same as (**a**) but for the inverse metawedge. The blue line in the top scan indicates the theoretical prediction of the turning point at the surface. Note that points A and B are used to measure the refraction angle associated with conversion, see text for more details.

The effectiveness of rainbow trapping, and in particular the spatial segregation by frequency, is analysed in Fig. 5a using experimental data. Scans of the wavefield are taken along the centreline of the top and the bottom surface and for each scanned position along the centreline a Fourier transform in time is calculated; the records are stopped before the main wavefront reaches the far right side to avoid spurious reflection. The analysis of the data focuses on the frequency band excited by the 3-cycles sinusoidal source. Figure 5a, besides the metawedge configuration, shows the magnitude of the Fourier coefficients as a function of position and frequency for the top and bottom surface. As predicted for the increasing metawedge, the signal at the bottom surface remains close to the ambient noise level with the most interesting phenomena happening at the top surface. The rainbow trapping is visible in the surface plot where lower frequency signal travels much longer through the metawedge. The trapping point (or equally turning point in Fig. 5b), is highlighted by a marked increase in amplitude (dark shaded stripe) and is exactly predicted using Eq. (1) combined with the metawedge geometry; the predicted trapping/turning point is shown by the blue line. As a detail, another patch where amplification occurs, is visible before the trapping point and further analysis shows that it is created by one of the flexural resonances of the rod. The flexural resonances result from the rod’s interaction with the Rayleigh waves. While this is an expected feature characterising the dynamics of the rods, previous studies^30, 36, 37^ have established that the dispersion properties of the metasurface are largely dominated by the longitudinal modes. These previous results are further confirmed here by the match between the blue line, calculated using the longitudinal resonance in Eq. (1), and the spectral analysis of the experimental data in Fig. 5a. When the surface waves are slowed down inside the metawedge, their wavelength become as short as the spacing between the resonators. At the same time, the amplitude is strongly amplified at the surface and in the resonator. For further clarification of this amplification, the bottom plot of Fig. 5a, shows the magnitude of the temporal Fourier coefficient for a frequency of 500 kHz (the central frequency of the input source) at different positions along the surface. To exclude any possible local effects induced by the presence of the resonators on the scanned area, the blue line results from an average over 5 different measurements each taken a few millimetres away from the centreline. Before interacting with the wedge, the amplitude of the Fourier coefficients is typically between 70 and 80 pm. In the trapping phase, this value increases dramatically reaching, on top of the resonators, magnitude $\gg$ than 300 pm. On the right side of the wedge nothing is transmitted with the signal dropping below the noise threshold. Thus, besides the trapping, the graded design can make possible ultrawide bandgaps.

Figure 4 reverses the orientation of the metawedge with the incident Rayleigh waves interacting with the longest resonators first, so the array is of decreasing height. To detect the conversion of Rayleigh into *S*-waves, the scanning procedure includes, as well as the top, also the bottom surface where the converted *S*-wave signal would be detected. To rule out any other boundary conversions that could create the signal we complement the experimental results with numerical simulations for the same propagation distance and geometry (videos are available as supplementary material). Figure 4 shows experimental detail on both the top and bottom of the sample, as in the rainbow trapping one observes in Fig. 4a and b that there is an incoming Rayleigh wave and it interacts strongly with the resonators. Unlike for rainbow trapping the signal is not reversed but propagates downwards through the sample to impact upon the bottom surface where it is picked up by the bottom scan. To demonstrate that this is not some artificial effect or spurious side reflection we show the accompanying numerical simulation in each panel with Fig. 4c showing the surface wave having been deflected in the *S*-wave. Figure 4d shows the bottom scan having picked up a surface wave along this lower surface of the block and some signal having been reflected and impacting back upon the top scan; these can be attributed to the finite nature of the sample, which is verified numerically.

We now move to Fig. 5b where the generation of *S*-waves is analysed in detail with the same method as the one used for the increasing metawadge. The frequency-position plot for the bottom scan, in this case, shows strong signals produced by the converted Rayleigh waves; the turning point is identified from the top scan and fits with the theoretical prediction. Because of the graded design, the transition between Rayleigh to *S*-waves happens very smoothly, without back reflection1, as opposed to Fig. 5a where the strong bandgap effect rules the behaviour and does not allow the wave to propagate any further. The turning point is still identified from Eq. (1), but the prediction is slightly less accurate because the conversion happens at the upper limit of the bandgap, and not exactly at the resonance, in contrast to the trapping. The prediction can be made accurate through a complex formula for the conversion given in a previous work^1, 30^. Nevertheless the physics emerges clearly such that the refraction angle *θ* generated by the conversion and measured between point A and B in Fig. 5b matches the Snell’s law prediction for Rayleigh to shear wave conversion in aluminium. By considering a shear wave velocity of *v*_*s*_ = 3100 m/s and a Rayleigh wave speed of *v*_*r*_ = 2900 m/s (both reference values for the aluminium used in the experiment), Snell’s law, cos(*θ*) = *v*_*s*_/*v*_*r*_, predicts an angle of ~21°. With a block thickness of 20 mm (Fig. 1a), the distance between points A and B (Fig. 5b) can be estimated to be about 40 mm; this yields an angle of ~25°. Given a wavelength between 5 and 8 mm for this frequency range and the lack of an abrupt discontinuity during the Rayleigh to S-wave conversion (contrary to the previous trapping case), the measure of the exact position of the points A and B can only be approximated, yet the resulting angle is in good agreement with Snell’s theory. As suggested by the Fourier coefficient at 500 kHz plot at the bottom in Fig. 5b, the conversion happens smoothly, with no clear sign of amplification (only small oscillations due to the rod’s resonance are visible), nor back reflection. After the conversion, the signal bounces up and down between the top and bottom surfaces as is reasonable to expect given the geometry of the aluminium block. The speed of propagation and the polarization suggest that waves in the elastic substrate are mainly propagating as *S* and Rayleigh phases with only little disturbances induced by the presence of the lateral boundaries. These reflections are clearly captured by both numerical simulations and surface scans of the vertical motion *u*_*z*_ (see also supplementary video material) and do not spoil the space-frequency analysis in Fig. 5.

## Discussion

We have conclusively demonstrated via experiments in the ultrasonic regime, complemented by theory and numerical simulations, that mechanical metasurfaces can be designed to create elastic rainbow trapping and to generate surface Rayleigh wave to bulk shear wave conversion. Both effects have great potential to obtain performance and functionalities hitherto unachievable. Because local resonance is at the origin of the elastic rainbow and the mode conversion, this technology can be tuned to the desired lengthscale through an appropriate engineering. Potential applications include, but are not limited to, resonant seismic barriers, structural vibration mitigation, micro and nano electromechanical components and the field of waveguiding of ultra and hypersounds. For instance the rainbow trapping leads to strong spatial localisation, segregated by frequency, of vibration energy ideally suited to energy harvesting applications and we envisage this opening up new possibilities. The waste of energy through vibration, and the potential to harvest it and generate efficiency savings is substantial. In addition, the spatial segregation by frequency is suggestive that this can be used as a wave filter, a shorter wedge removing all frequencies within a band, and as a wave sensor to pick out specific components of the wave spectrum. These ideas are closely related to the acoustic black hole concept where an area with exponentially increasing refractive index (>1) attracts and ultimately traps waves^38, 39^. In practice, this technology can be applied only to elastic plates with applications similar to those discussed here: With the introduction of the metawedge, a resonant elastic black hole can be envisaged. Mode conversion, allowing surface waves to be diverted into bulk waves, is also a powerful concept creating the existence of regions of surface wave silence or equivalently protection from surface waves; this has clear application to many vibration related problems in mechanical engineering (high speed and high precision manufacturing), civil engineering and geophysics where the area of seismic metamaterials and seismic protection devices is very topical^36, 40^.

## Methods

### Metawedge fabrication

The sample was CNC milled out of a solid block of aluminium using a 1 mm diameter cutter (machining time: 22 h). The finished sample was approximately 300-mm × 30-mm × 20-mm. The metawedge structure consists of resonators with 0.5 × 0.5-mm cross-section and of heights graded from 0.5-mm to 3-mm over 60-mm along the block and spaced by. 1.5-mm placed midway along the block and covered the full width of the block. The width of the block was chosen so that the ultrasound could be excited reasonably uniformly across the full width. The thickness of the block (20-mm) was chosen to be thick enough to render any dispersion (due to Lamb waves in the finite block) irrelevant so the surface wave could reasonably be treated as a Rayleigh wave at the frequency used in the experiment (500 kHz/*λ*~6-mm). The length of the block was chosen so there was ample space to fit the transducer used at either end of the block and differentiate all the separate echoes and signals received in time. The sample was milled flat on the top and bottom surfaces and held in a jig that allowed the ultrasound to be observed either on the top or bottom surface and with the ultrasound propagating from either end of the block.

### Experimental set-up and measurements

The sample surface was smoothly milled but optically rough and a rough-surface capable optical detector (Bossanova Tempo-10HF) was used as a detector. The detector was used in absolute calibration mode so that the signal amplitude measured at each point was directly proportional to the surface amplitude regardless of the optical return at that point. The ultrasound was excited using a Panametrics Videoscan V414 0.5 MHz plane wave transducer with a 65° polymer wedge to couple the longitudinal wave of the transducer into a Rayleigh wave on the sample. The transducer and wedge were glued to the sample using phenyl salicylate which provided good coupling and long term stability while allowing easy removal and reattachment of the transducer. The transducer was driven by a Ritec RPR-4000 programmable pulser using a 3-cycle sinusoidal burst at 500 kHz with an amplitude of 195 V peak-to-peak and repetition rate of 500 Hz. At this repetition rate, all echoes from the previous pulse were able to die out before the next measurement. The signal was captured using a digital storage oscilloscope (Lecroy Waverunner104Xi-A) and averaged 100 times before transfer to a desktop computer. The sample was mounted on scanning stages with a movement range of 300-mm × 50-mm and nominal positional accuracy of 1-*μ* m allowing the full surface of the sample to be scanned. Scanning was performed at a rate of ~2 points/second. This scan rate was primarily limited by the settling time of the optical detector between positions on the sample surface.

### Numerical simulations

The propagation of surface waves in a 3D halfspace is a common problem in numerical elastodynamics and modeled applying traction free conditions on the top-surface and on the micropillars. In order to compare the simulations quantitatively to the experimental results, traction free conditions are also applied to the sides of the aluminium block in the simulation thereby representing the aluminum block sides of the experimental sample. Only the side closest to the source is supplied with absorbing boundaries (perfectly matched layers^41^) to suppress the backward reflection of the Rayleigh wave. In the actual experiment, this reflection is also not present because the plastic wedge between the transducer and the aluminium block (Fig. 2) only allows Rayleigh waves to propagate forward towards the metasurface. As in the experiment, the plane Rayleigh wave front is obtained with a 3 cycle sinusoidal source time function and 40 point source synchronously triggered on the top surface at a distance of 6 cm from the first row of resonators. The 3D time domain simulations have been carried out using SPECFEM3D a code that solves the elastic wave equation using finite difference in time (Newmark scheme) and the spectral element method in space (5^*th*^ order polynomials)^35^. The parallelization is implemented through domain decomposition with MPI and the mesh is made of hexahedra elements and it is generated using the commercial meshing software CUBIT. Given the high quality factor of aluminium ($\gg 1000$), attenuation has not been considered. Simulations are then run on a parallel cluster (Froggy at University of Grenoble) on 256 CPUs. Approximately a total of 4000 core-hours have been used for this study. 3D plots have been generated with Paraview while 2D plot with Matlab and Matplotlib.

## Electronic supplementary material

Rayleigh to S wave experimental

Rayleigh trapping experimental

Rayleigh to S wave numerical
